# Priming for Life: Early Life Nutrition and the Microbiota-Gut-Brain Axis

**DOI:** 10.3390/nu13020423

**Published:** 2021-01-28

**Authors:** Anna Ratsika, Martin C. Codagnone, Siobhain O’Mahony, Catherine Stanton, John F. Cryan

**Affiliations:** 1 APC Microbiome Ireland, Biosciences Institute, University College Cork, Cork T12 YT20, Ireland; anna.ratsika@ucc.ie (A.R.); martin.codagnone@ucc.ie (M.C.C.); somahony@ucc.ie (S.O.); catherine.stanton@teagasc.ie (C.S.); 2 Department of Anatomy and Neuroscience, University College Cork, Cork T12 YT20, Ireland; 3 Department of Psychiatry and Neurobehavioural Science, University College Cork, Cork T12 YT20, Ireland; 4 Teagasc Food Research Centre, Moorepark, Fermoy P61 C996, Ireland

**Keywords:** nutrition, early life, microbiota-gut-brain axis, brain development, breast milk, infant formula

## Abstract

Microbes colonize the human body during the first moments of life and coexist with the host throughout the lifespan. Intestinal microbiota and their metabolites aid in the programming of important bodily systems such as the immune and the central nervous system during critical temporal windows of development, with possible structural and functional implications throughout the lifespan. These critical developmental windows perinatally (during the first 1000 days) are susceptible timepoints for insults that can endure long lasting effects on the microbiota-gut-brain axis. Environmental and parental factors like host genetics, mental health, nutrition, delivery and feeding mode, exposure to antibiotics, immune activation and microbiota composition antenatally, are all factors that are able to modulate the microbiota composition of mother and infant and may thus regulate important bodily functions. Among all these factors, early life nutrition plays a pivotal role in perinatal programming and in the modulation of offspring microbiota from birth throughout lifespan. This review aims to present current data on the impact of early life nutrition and microbiota priming of important bodily systems and all the factors influencing the microbial coexistence with the host during early life development.

## 1. Introduction

Starting from the first moments of life, the human body is colonized by a wide variety of microorganisms [1,2] that coexist with the host for mutual beneficial purposes [3]. These microorganisms colonize the skin and various mucosal cavities (oral, nasal, vaginal and pulmonary), yet the vast majority of them are located within the gastrointestinal (GI) tract and are termed as the intestinal microbiota [4]. The composition of the intestinal microbiota is believed to largely assemble after birth influenced by early life events such as delivery mode [1], early life nutrition [5], and antibiotic exposure [6]. The microbiota continues to expand and develop in accordance with the needs of the host across the lifespan.

The microbiota has been found to influence not only at a local level with respect to the intestinal microenvironment but also beyond the GI tract, implicating the physiological and structural aspects of the central nervous system (CNS). The communication pathways which enable the interaction of intestinal microbiota with the CNS of the host is described as the microbiota-gut-brain axis [7,8,9,10]. Although the gut-brain axis was initially a target for research on hunger, satiety and digestion [11,12] most recent studies have focused on cognition and behaviour; the impact of psychological stress on GI motility, permeability and secretion, as well as the effect of afferent neuronal fibre stimulation in the gut on certain psychopathologies [7,13]. The pathways of the gut-brain axis have emerged as novel targets for mental health conditions, as well as for obesity and GI disorders including irritable bowel syndrome (IBS) [9,14,15].

To date, the majority of data on the microbiota-gut-brain axis has been gathered from studies using animal model systems. Indeed, various germ-free (GF) animal studies have shown that lifelong absence of microbiota not only alters gut physiological functions, but also the behaviour of those animals [16,17,18]. Similar findings are now appreciated regarding the effects of antibiotic-induced microbiota depletion on intestinal permeability, behaviour and cognitive functions [19,20,21]. There are increasing studies in humans, both in early life and throughout the lifespan validating such findings [9,22,23,24].

Despite intensive investigations, the mechanisms underlying the communication pathways between intestinal microbial systems and the host remains rather elusive. Moreover, the full implication of such interactions on health and disease in critical temporal windows across the lifespan are still being unravelled. The impact of early life nutrition on the composition of the gut microbiota, the microbiota-mediated priming of CNS and immune system and the response of the host during this crosstalk is intricate and complex. The aim of this review is to summarize recent knowledge and highlight the effects of early life nutrition on the gut-brain axis development, the shaping and maturation of the intestinal microbiota and the dialogue among these stakeholders during critical periods of neurodevelopment.

### 1.1. Disruption of the Microbiota-Gut Brain Axis

**Figure 1:**
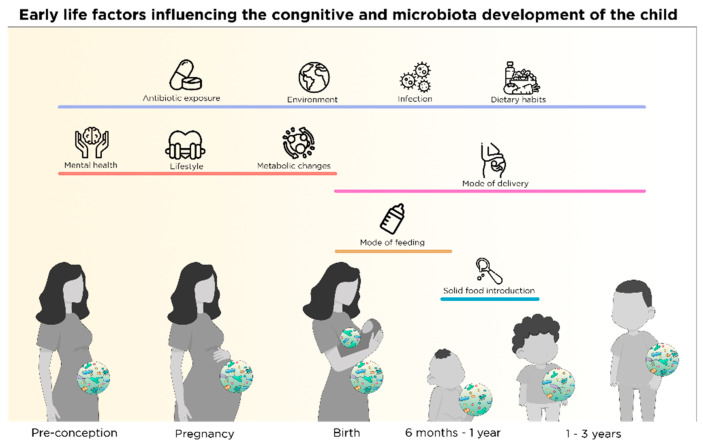
Early life factors influencing cognitive and microbial development of the child from preconception throughout the first 3 years of life; maternal mental health, lifestyle and metabolic changes affect fetal development. Antibiotic use, infections, environment, dietary habits and mode of delivery impacts the maternal health status, the fetal development during pregnancy and the microbiota and cognitive development of children from birth to at least 3 years. Mode of feeding in early life and solid food introduction influence the microbial and cognitive development of the offspring from birth up to at least 3 years of age.

Homeostasis of the intestinal microbial environment is likely to be affected multiple times across the lifespan of the average individual due to antibiotic usage [25], inflammation [26], ageing [27], psychological stress [28], nutrition and lifestyle choices [29], as well as other environmental factors (i.e., smoking, pollution, mode of birth) [1,30,31,32,33] (see Figure 1). Early life modulation and priming of the microbiota has been found to influence brain health and disease state later in life [34]. For instance, alterations in bacterial composition in the gut during early life have been correlated with behaviours associated with autism spectrum disorder [35,36,37]. Moreover, gut microbiota perturbations (i.e., via antibiotic exposure) in early life has been associated with a higher risk of mental illness such as anxiety and depression in humans [38], among others [39]. However, there are also epidemiological studies showing limited effects of antibiotic exposure in early life on mental health outcomes [40] indicating there is more at play than just altered composition of the microbiota.

**Figure 2:**
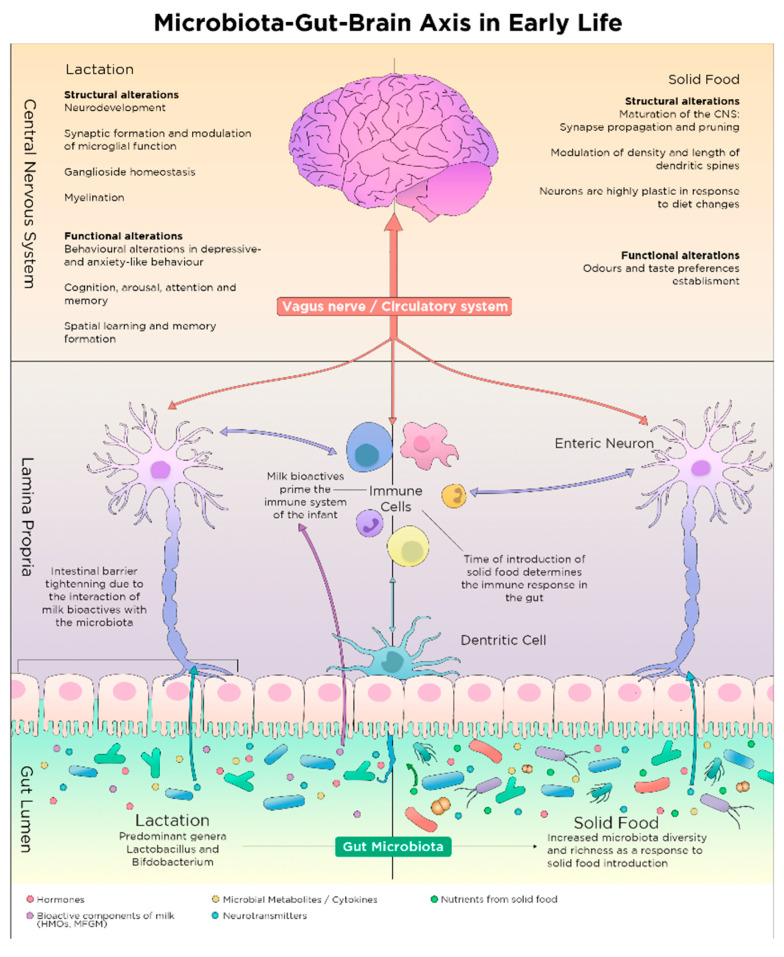
The effect of early life nutrition during lactation and solid food introduction on the gut microbiota development in the intestinal and the central nervous system; Microbes become more diverse and mature with solid food introduction compared to lactation. Microbes and metabolites in the gut lumen (hormones, neurotransmitters, microbial metabolites, cytokines and nutritional components from milk or solid food) affect the host physiology via the gut-brain axis; they modulate the epithelial barrier, the homeostasis in the lamina propria and the brain. Nutrients, metabolites and microbes in the gut lumen signal to dendritic cells and the enteric neurons which subsequently exchange signals with the immune the circulatory and the central nervous system. The vagus nerve, the immune and the enteric nervous system are all pathways of communication among the microbes, the gut and the brain.

Despite the underlying mechanisms of the intercommunication along the microbiota-gut-brain axis remain incompletely understood, there is a variety of potential trajectories (some of which are depicted in Figure 2) through which the intestinal microbiota may influence the CNS [41,42]. Pathways associated with the reciprocal exchange of signals from brain and microbiota include the vagus nerve, the hypothalamic-pituitary-adrenal (HPA) axis, the immune system, as well as neurotransmitters and metabolites with neuroactive properties produced by the microbiota in the intestines [9,41]. Nutritional components are known to impact on these pathways and are able to modify offspring development via these multiple trajectories providing links between microbiota, brain development and nutrition.

### 1.2. Nutrient-Microbiota Interactions and Gut-Brain Axis

The intestinal surface interacts with a plethora of microorganisms, as well as with food in the form of macro- and micronutrients that constitute substrate for host cells and microbes [43]. Nutrients and metabolites are sensed by the intestinal epithelial cells, initiating a hormonal cascade that eventually leads to nutrient absorption and transportation via the circulatory system to various tissues within the body [43]. Different pathways are activated in order to digest the various nutrients in the gut [44]. For instance, fatty acids in the gut are sensed by G-protein coupled receptors that mediate the production of incretins—gastrointestinal hormones—such as GLP1 that promotes insulin release from pancreatic cells [45].While nutrients interact with microbiota, important secondary molecules are released to be later absorbed by the host. For example, prebiotic non-digestible dietary fibres are fermented by microbiota which release secondary metabolites that regulate important processes in the human body [46].

One category of highly potent by-products of fibre fermentation by the gut microbes are short-chain fatty acids (SCFAs), which include acetate, butyrate and propionate [47]. These metabolites are absorbed in the intestine and are able to modulate the CNS [48,49] and the immune system [50,51]. Apart from their ability to cross the blood-brain barrier (BBB) and reach the brain, modulating structural and functional aspects of the CNS [47], SCFAs produced by commensals also affect the immune response via regulation of dendritic and T-cell function, as well as via inhibition of cytokine production [52] which affect brain development. It is becoming clear that the roots of the microbial and nutritional reciprocal relationship within the gut-brain axis and the balance between health and disease lie in the early life priming of these bodily systems supported by the initial nutrient-microbiota crosstalk [53].

## 2. Early Nutrition-Microbiota Crosstalk in Sensitive Time Windows of Development

Nutrition holds a central role in the early life maturation of many tissues within the body, with both short- and long-term effects on development of the infant, in an organ-, time- and intervention-dependent fashion, which is called nutritional programming [54]. Nutritional programming refers to the ability of highly potent molecules that are normally present in the diet or are *de novo* synthesized in our body to modulate and support early life development. There are multiple nutrients with epigenetic potential that are present in the diet or produced via microbial metabolism in the human gut [55]. B complex vitamins, SCFAs and polyphenols are among nutrients or microbial metabolites that are known to exert epigenetic effects on the host and affect fetal programming in sensitive time frames of development [55].

These sensitive time-periods are temporal windows of developmental opportunity that, if missed, alterations in growth and normal functions of body systems are irreversible [56,57]. These periods include pre-conception, pregnancy, peri- and early postnatal life that are characterized by rapid changes in maturation of neuronal, immune, endocrinal and metabolic processes [58,59]. The quality and quantity of food received during these sensitive periods are crucial indicators of weight gain and metabolic regulation [54,60], as well as CNS development, microbiota composition and immune system priming of the individual across the lifespan [42,56,61,62,63].

### 2.1. Nutritional and Microbial Regulation in Pre-Conception

Parental nutrition and health status prior to conception are crucial for the appropriate structural development of the CNS of the offspring (see Figure 1). Certain micronutrients with epigenetic potential, such as folate, are recommended to women who plan to get pregnant or are already in early stage of pregnancy in order to prevent infant neural tube defect and other congenital malformations [64]. However, vitamin kinetics and drug-microbiota-nutrient interactions should be considered while receiving supplementation. For instance, certain bacteria in the gut are able to produce *de novo* folate as a secondary molecule of their metabolism [65]. Furthermore, exposure to oral contraceptives or anti-epileptic drugs might decrease the availability of folate in the body [66,67], thus risking congenital malformations in the case of pregnancy.

Increasing research suggests that environmental influence on parental health can modulate functional aspects of infant development, such as offspring’s behaviour later in life [68,69]. Recent preclinical data revealed that paternal immune activation due to infection modulates offspring behaviour via epigenetic regulation of the paternal reproductive cells, with persistent inheritable potential for at least two generations [70]. Modulation of the maternal immune system by environmental factors, before and during pregnancy, is able to induce persistent alterations in offspring health from early life throughout adulthood [71,72].

Expanding data point to the existence of critical temporal windows before conception, regarding parental nutrition and microbial metabolites shaping the immune system of the offspring [63,73,74,75]. In rodents, reduced parental exposure to bacteria prior to conception could lead to allergic disease in the offspring via epigenetic regulation of immunomodulatory genes, that passes to the next generation [72]. Nutritional-microbial crosstalk is able to exert inheritable changes in the germline and holds a central role in fetal programming of central nervous and immune systems.

### 2.2. Nutrition-Microbial Input on Neurodevelopment in Pregnancy

The brain is subjected to multiple structural and functional, time-specific changes during gestation such as axonal growth [76], synapse formation and dendritic and axonal arborization [77]. After neuronal differentiation, synaptic connections among neurons continue to develop during gestation [78]. During the early phase of the cascade of those time-dependent and strictly controlled events, disruptions can determine the fate of brain function later in life [78]. Therefore, this period is crucial for structural (brain connectivity) and functional (cognitive development and behaviour) outcomes in the brain [79]. During pregnancy, maternal factors (diet, lifestyle, mental health, antibiotic use) as well as environmental factors (infection, air pollution, tobacco or radiation exposure) impact the health of the pregnant mother [80,81] and affect fetal development via transplacental signals, including signals from maternal intestinal microbes and nutrients [53] (see Figure 1).

#### 2.2.1. Maternal Nutrition and Fetal Neurodevelopment

Maternal nutrition during pregnancy can modulate some of the multiple structural and functional changes that are happening in the offspring brain perinatally [78]. In fact, nutrient intake influences micro- and macro-structural aspects of the brain during various time points of brain development [82,83] via regulation of neurotransmitter pathways, synaptic transmission and signal-transduction pathways [61,84]. Such nutrients are the ω-3 polyunsaturated fatty acids which have a known effect on brain plasticity, cognition and brain health via regulation of hippocampal BDNF in rodents [82]. In humans, ω-3 fatty acid consumption in early life is associated with improved cognition, while decreased levels of ω-3 fatty acids have been found in the brain of individuals with mental health and neurodegenerative conditions [82,83].

Nutrient deficiencies in the mother during pregnancy may result in abnormal neurodevelopment of the fetus, in humans, which leads to related adversities in the adult offspring [85,86]. For instance, molecules affecting one-carbon metabolism like folate, choline and betaine, have known epigenetic potential in nervous system development of the offspring [87]. All of these molecules are present in green leafy vegetables, as well as in beets, wheat and seafood [88], while folate can be synthesized as a secondary metabolic product of certain bacteria in the gut [65]. Optimal nutrition is required to support brain macro- and micronutrient requirements for its maximum developmental potential [83].

#### 2.2.2. Maternal Nutrition and Offspring Gut Microbiota Development

Dietary habits during the period of gestation can shape the maternal intestinal microbiota [81,89] which result in altered microbial metabolites in the maternal intestines. For instance, dietary fibres in the maternal intestines are fermented by maternal microbes with the subsequent release of microbial by-products known as SCFAs. The SCFAs are able to pass from the gut lumen to the circulation and travel to the fetus via the placenta [90]. SCFA and other microbial metabolites from the maternal intestines, are able to imprint in utero development with possible health outcomes on the offspring across lifespan [53,90].

Maternal weight and obesity have been associated with alteration in composition of the gut microbiota, with obese individuals displaying altered microbial profiles compared to normo-weight individuals [91]. Microbial signatures of overweight and obesity have been found to pass from mothers to infants, with infants from obese mothers obtaining distinct microbiota profile compared to infants from normo-weight mothers [92,93]. Moreover, stool microbiota analysis has shown decreased abundance of the *Bifidobacterium* group in babies of overweight mothers compared to babies born to normo-weight mothers [92] emphasizing the possible role of *Bifidobacterium* on weight development and weight control of the infant.

In a cohort of Hispanic mother-infant pairs, low levels of *Bacteroides* have been found in neonatal meconium samples from infants with mothers exposed to a diet with increased fat content (>40% of daily intake) during pregnancy, with those low levels of *Bacteroides* persisted until 6 weeks of age [81]. The *Bacteroides* genus, part of the commensal bacteria in the gut, is present in low amounts in the infant gut right after birth and it becomes gradually more abundant after solid food introduction [94]. Low counts of *Bacteroides* in adulthood have been previously associated with obesity and altered metabolic capability of the host [94].

In preclinical studies, maternal dietary manipulations during pregnancy can modify the offspring microbiota composition [95]. For instance, in rodents, microbial diversity and composition of pups originating from dams exposed to a high-fat diet (HFD) (60% of daily intake) during pregnancy was altered, with reduced abundance of *Lactobacillus* *reuteri*, *Bifidobacterium pseudolongum* and *Bacteroides uniformis* present in the faeces of pups originating from dams exposed to HFD compared with pups originating from dams exposed to normal chow diet (13.4% of daily intake) [96]. Additionally, microbiota composition changes in the offspring as a result of maternal HFD during pregnancy were accompanied by impairments in social behaviour of the pups tested by the reciprocal social interaction and the three chambers social interaction tests [96]. Moreover, maternal high-fibre diet (21% wt/wt, 1:1 ratio of oligofructose and inulin) in rats during pregnancy and lactation modified the gut microbiota of the dam and the offspring [97], probably via a combined effect of vertical transmission of maternal microbiota during birth, and breast milk microbiota in the suckling pups.

Apart from maternal nutrition during pregnancy, there are other factors modulating the offspring’s intestinal microbiota composition in early life such as mode of delivery, type and duration of feeding (see Figure 1) which are discussed in separate sections below. Maternal antibiotic exposure perinatally, as well as infant antibiotic exposure postnatally are strong modifiers of offspring microbiota composition and have been recently discussed elsewhere [6].

#### 2.2.3. Maternal Microbial Signatures in Pregnancy Prime Fetal CNS

The composition of the maternal gut microbiota varies dramatically in response to hormonal, immunological and metabolic changes that take place during pregnancy [98]. It is known that there are tight molecular and cellular interactions between mother and fetus via the placenta [99]. The maternal microbiota produces compounds that are transported to the fetus via transplacental pathways leaving metabolic signatures to the developing fetus [98] and these prenatal microbial signatures can tip the balance between health and disease [53,100].

For the past decade, scientists examined controversial data regarding the possibility of a non-sterile environment during the course of gestation [75,101,102]. Recently, sparse but viable microbiota have been identified in the fetal gut at mid-gestation in humans [75]. However, this clinical study has been recently heavily criticised by other researcher of the field [103,104] who claim that contamination of the samples during analysis is the most probable explanation for the bacteria found in the fetal gut. Researchers who analysed meconium (the first faecal sample of the new-born) found low amounts of viable bacteria present but confirmed that the main bacterial colonization of the gut happens at the time of birth [105]. Even though it is difficult to assess the ‘in utero sterility hypothesis’ due to possibility of external contamination of samples, it is believed that microbes and their metabolites in the maternal intestines influence in utero CNS development and function [106].

The assembly of fetal neuronal circuits can be perturbed in response to maternal and environmental microbial disruptions during pregnancy. Recent data suggest that depletion and partial reconstitution of the maternal microbiota during pregnancy modulate fetal thalamocortical neurodevelopment in rodents -via transplacental metabolic signals-which is linked to sensorimotor behaviour and pain perception postnatally [106]. Additionally, microbial signatures of maternal stress during pregnancy are able to pass to the next generation, modulate hippocampal development and gut function in adult male offspring in mice [107]. Epidemiological studies focused on maternal infection has shown that maternal immune activation (MIA) perinatally is associated with impaired fetal brain development and higher risk of acquiring psychiatric disorders in adulthood [108,109]. Therefore, changes or perturbations of the maternal microbiota during gestation primes fetal neurodevelopment during this critical period and might determine cognitive, sensory and behavioural function of the offspring throughout the lifespan. Data present in the current review are referred to term-born infants (unless otherwise specified), as the effect of premature birth on the microbiota [110] and the brain development have been discussed elsewhere [111].

### 2.3. Mode of Birth and Microbiota

#### 2.3.1. Vaginal Delivery and Vertical Transmission of Microbiota

Today it is understood that the initial microbiota inoculation begins at birth while the fetus passes through the birth canal and is exposed to maternal vaginal and faecal microbiota [1]. This constitutes the first moment leading to extensive microbiota colonization of the neonate; multiple studies have reported the impact of delivery mode on the neonate’s first microbial exposure [1,101,112]. Dominguez-Bello and colleagues demonstrated that there is vertical transmission of the maternal microbiota to the new-born in vaginal delivery, meaning that the neonatal microbiota resemble the maternal vaginal and faecal microbiota [1]. These vertically transmitted microorganisms have the potential to affect the development of intestinal microbiota in the offspring during early life and may relate to host health later in life [113].

#### 2.3.2. Caesarean Section and the Missing Microbes

During Caesarean-section (C-section) the infant is not exposed to the maternal vaginal and faecal microbiota and the vertical transmission is disrupted [1]. Interestingly, it is understood that the intestine of C-section delivered new-borns is predominantly colonized by bacteria present on the skin and in the environment [1,112]. In contrast with vaginal delivery, the microbiota of C-section babies was found to display decreased diversity and richness [114], with C-section being associated with significantly lower levels of bacterial genera essential to brain development such as *Bifidobacterium*, *Lactobacillus* and *Bacteroides* [112,115], and higher levels of *Staphylococcus* [1].

However, emergency C-section impacts the microbiota differently compared with elective C-section, due to the fact that the neonate is partly exposed to the birth canal in the early stages of labour [116,117,118]. In the case of emergency C-section, the newborn microbiota resembles more closely the composition of vaginally-delivered babies [119]. Interestingly, elective and emergency C-section have been also found to alter microbial diversity and richness in breast milk [120,121]. Even though depletion of specific microbiota in breast milk could be partly explained by administration of antibiotics to the mother during C-section procedures, it has been recently proven that C-section is an independent modifier of breast milk microbiota [121] indicating this can also contribute to the differences seen between babies born via different modes.

Mode of delivery is a strong modifier of the relationship between maternal perinatal nutrition and postnatal infant microbiota composition development [81]. In a clinical study pre-pregnancy maternal weight impacts infants’ microbiota composition in babies born vaginally to obese mothers, but not via C-section [93]. The disruption of vertical transmission in this case might be beneficial for the microbiota composition development of the offspring and prevent the ‘obese’ microbial signatures that could have passed with vertical transmission and could relate to offspring health later in life.

Although mode of delivery has a widely known impact on the new-born microbiota, its exact influence on microbiota shaping throughout the lifespan is still rather controversial due to confounding factors (i.e., pre-existing conditions such as pre-eclampsia, emergency or elective C-section and antibiotic use during surgery) [119]. Even though the impact of being born via C-section leaves microbial signatures up to four years of age in a recent human cohort [118], other studies claim that the microbial composition of C-section individuals recovers with of time, displaying a transient effect [112].

Ways of modulating gut microbiota such as faecal microbiota transplantation (FMT) in both clinical and preclinical context, are being studied. It is evident that microbiota interventions like FMT could reinstate gut microbiota composition of individuals with clinical or subclinical intestinal conditions such as diarrhoea, IBD and intestinal infections. [122]. Most studies on early life FMT are focused on the treatment of symptoms of IBD or recurrent infections of *C. Difficile* [123]. The concept of FMT in C-section individuals is novel, in order to reinstate the gut microbiota of those individuals to resemble more the microbes of vaginally-delivered infants. Indeed, such an approach was taken in a recent small clinical study where seven C-section delivered new-borns received FMT shortly after birth from their mothers’ faecal microbes [124]. The FMT was able to restore the microbial differences between C-section and vaginally-born infants up to 3 months of age [124]. Future studies are needed to explore the feasibility and safety of such approaches in the future.

Most developmental studies investigating the mode of delivery focus on bacteria but, recently scientists are shifting their interest on the effect of birth mode to the gut virome (the assemblage of viruses present in the intestine) and phageome (the bacteriophage community present in the intestine) [125,126] which may also contribute to the future health of the offspring.

#### 2.3.3. C-Section-Related Risks and Adversities

Early life disruption of microbiota by C-section has been associated with some well-known disorders in childhood and adulthood. In clinical studies, C-section has been correlated with immune disorders including asthma and allergies [127,128,129], along with obesity [130] and type 2 diabetes [114,131]. In human epidemiological studies there have been links between C-section and school performance [132,133] but this has not been reproduced in other datasets [134]. Moreover, a small association between planned C-section and visual-spatial cognitive delay in childhood has been reported [135]. In pre-clinical studies, C-section is linked to neurodevelopmental structural changes that are accompanied by early life behavioural alterations on infant vocalization during maternal separation [136,137] as well as anxiety-like behaviour throughout lifespan [138].

It could be hypothesized that behavioural effects due to differential microbial colonization as a result of C-section, are possibly extended to later stages of life, affecting multiple aspects of social behaviour. However, more studies are needed to investigate the long-term microbiota changes and behavioural effects of delivery mode, with shifting focus to the missing microbes in C-section delivered individuals. Targeting the behavioural effects with probiotic intervention strategies to potentially rescue behavioural deficits is a rather promising avenue for those individuals.

### 2.4. Shaping of Microbiota Composition and Neurodevelopment via Postnatal Early Life Nutrition

The brain is highly metabolically active in early life and its energy expenditure accounts for half of the total daily resting energy metabolism [61]. At full term birth, the brain weighs around 350 g, representing only around 10% of infant’s body weight, while at one year of age the brain weight reaches 925 g which accounts for 70% of the adult brain weight (~1300–1400 g) [78]. The high brain-weight to body-weight ratio, anatomical, structural and functional brain changes, as well as the demanding metabolic rate of the CNS, are indications that CNS development in critical time windows is sensitive to energy and nutrient availability, which is believed to define the fate of development, physiology and mental health later in life [61].

#### 2.4.1. The Microbiota Expansion and the First Food after Birth

The new-born gut is mainly colonized by various species of *Bifidobacterium*, that are also highly abundant among the commensals in the maternal breast milk. The offspring gut microbiota modulates multiple levels of development, almost immediately following birth. For instance, certain bacteria have been shown to stimulate gene expression of tight junctions in mice, boosting the closure of the gaps between epithelial cells and promoting gut barrier maturation postnatally, that is known to be immature in the early steps of life [139]. In the intestine, *Bifidobacteria* have been found to boost gut barrier function, and improve intestinal disease outcome by decreasing the intestinal permeability in rodents [140]. In preclinical models, *Bifidobacterium* can alter structural characteristics of the CNS and modify neurodevelopment in early postnatal life by promoting synaptic formation and microglial function postnatally [141], while in later stages of development these bacteria have been found to rescue behavioural deficits such as anxiety- and depressive-like behaviour [142,143]. Collectively, all these findings highlight the importance of *Bifidobacterium* in regulating multiple aspects of development following microbial colonization at birth.

Soon after birth, microbiota in the infant gut is nurtured and shaped by the dietary and bioactive components of milk that are discussed below (see Section Bioactive Components of Breast Milk and Figure 2). Breastfeeding, breast milk from donors and infant formula are the three options currently available for early life nutrition [144]. The nutritious and bioactive interchange of the first food with microbiota in the infant gut inextricably and constantly modulate the microbiota composition of the infant which might relate to several aspects of infant development [145]. Early life nutrition determines the fate of microbial colonization, as well as the development of the GI tract, the immune and the central nervous system of the infant [146].

#### 2.4.2. Breastfeeding and Composition of Breast Milk

##### Breastfeeding

Breastfeeding is considered the gold standard for infant nutrition as it is tailored to provide various micro- and macronutrients for the demanding development of the new-born in a time-dependent manner [145,147]. Beyond its nutritional benefits, breastfeeding exerts other protective benefits for the developing child; it is known to enhance neurodevelopment and to boost the immune system in early life, but is also associated with decreased risk of childhood obesity and type 2 diabetes [146,148]. Furthermore, exclusive breastfeeding is highly recommended for the first 6-months of life, it is convenient and inexpensive, and it strengthens the bonding between mother and baby [149,150].

##### Maternal Characteristics, Breast Milk and Child Development

Breast milk composition is highly affected by maternal characteristics such as mental health [151], nutrition and lifestyle choices [152,153], protein intake, the return of menstruation, and nursing frequency [154]. Maintaining a healthy maternal body and mind during lactation influences the quality of breast milk which in turn is crucial for infant development [155].

The perinatal period is a time that women are highly sensitive to psychosocial and psychological stress as well as anxiety, which may induce perinatal depression to susceptible mothers [156]. Maternal exposure to stress and depression perinatally can lead to changes in mood, inadequate food consumption and lifestyle choices (lack of physical activity, alcohol consumption, smoking and substance abuse) and might affect lactation and the quality and composition of the breast milk [80,151].

Changes in breast milk composition due to maternal psychopathologies could eventually impact the neurodevelopmental outcome of the infant [151]. For example, in humans, postpartum depression has been found to modulate the concentration of polyunsaturated fatty acids (PUFAs) in breast milk, which in turn, are associated with increased risk of mental health conditions of the offspring [157,158]. Moreover, maternal mental health conditions (such as perinatal stress and postpartum depression) were assessed in an African population by Perceived Stress Scale questionnaires on days 3, 9, and 14 postpartum. Breast milk and saliva were collected on the same days as the questionnaires. Positive correlations were found in this study, between maternal stress and breast milk interleukin-8 at day 3, and with macrophage inflammatory protein-1-alpha (MIP-1α) at day 14 postpartum [159]. Cytokines in breast milk are able prime the CNS, immune system [160] and might modulate the microbiota composition of the new-born during critical temporal windows of development.

During the demanding period of lactation, the maternal energy requirements are increased; even though this depends on the stage of lactation [150], the general recommendations for the lactating mother (normo-weight) during exclusive breastfeeding, suggest consumption of extra 650 kcal/day [153]. However, if those extra calories are not consumed, the energy drawn from internal maternal stores to maintain lactation [150]. Breast milk composition is affected by maternal energy consumption and food choices during lactation [152,153]. However, the ability to produce milk is independent of certain maternal factors such as maternal weight, BMI, body composition and gestational weight [161]. Nowadays, various popular diets like vegetarian and vegan, might lack certain vitamins and calcium and therefore lactating women with these dietary choices might require supplementation during the breastfeeding period [150,162].

Non-maternal characteristics but situations linked to pregnancy such as premature birth (delivery before the beginning of the 37th week of gestation) cause changes in human milk composition. Breast milk of mothers with premature delivery contained significantly higher amounts of protein and immunological components compared to milk from full-term mothers [163]. Interestingly, protein content of the human milk was associated with the maternal BMI but not the maternal diet [154]. The composition of the human milk is also affected by the breastfeeding frequency, as the higher the nursing frequency the higher levels of lactose and lower levels of fat and protein are found in human milk [147,154].

**Figure 3:**
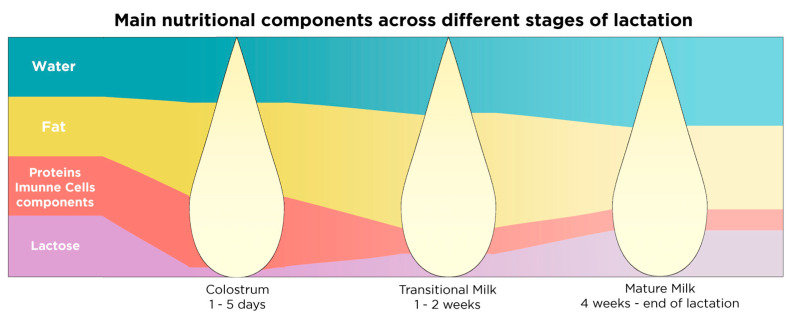
Main nutritional and bioactive components in the different stages of lactation. The quantity of the main nutritional components stays consistent in mature milk, after the 1st month of lactation until the cessation of breastfeeding.

Human milk composition is dynamic, varies within a feeding, diurnally, over the different stages of lactation, and between mothers [151]. Breast milk composition changes dramatically over the first month of life in order to match the new-born’s needs in macronutrients and immunity, but only subtle changes are identified in breast milk composition after the first month of lactation [147]. So far, three separate stages of lactation have been determined according to the composition of breast milk, which are summarized in Table 1 and Figure 3.

##### Macro- and Micronutrient Composition of Breast Milk

Breast milk is composed of a distinct combination of constituents that leads to specific metabolic and physiological responses in children, regulating intestinal function, immunity and brain development [60]. The components of human milk are derived from three primary sources: maternal diet, maternal macro- and micro-nutrient stores and the production of nutrients in the lactocyte (milk-producing cell located at the mammary gland) [147].

Macronutrient composition of breast milk varies within mothers and across lactation and it highly depends on maternal diet [152]. However, macronutrient composition differs according to the stage of lactation with early milk being richer in protein and fat compared to term milk [147]. Micronutrients in human milk such as vitamins and minerals depend highly on maternal diet and internal stores [152]. The most abundant macro- and micronutrients in breast milk can be found in Table 2.

##### Bioactive Components of Breast Milk

Multiple bioactive milk components have been found to modulate the immune system via gut microbiota [63,164], enhance CNS development and promote gut health in early life [151,165]. These promiscuous molecules are produced in the mammary gland, or by cells present in the human milk like immune cells, while some are transported into the mammary gland via circulation. Three of the most abundant bioactive components in human breast milk including milk fat globule membranes (MFGM), human milk oligosaccharides (HMOs) and breast milk microbiota are summarized in Table 3. Some bioactive components of breast milk, like the MFGM, may act as macronutrients too, providing the infant with energy or acting as building blocks for growth of various tissues.

##### Milk Fat Globule Membrane

Milk fat globules (MFG) are lipid droplets surrounded by a phospholipid tri-layer and are secreted locally by the mammary lactocytes [165,189]. The tri-layer surrounding the MFG, called milk fat globule membrane (MFGM), has a unique complex structure consisting of phospholipids, glycolipids, carbohydrates and proteins, as well as associated transmembrane growth factors on its surface, all of which have bioactive properties [165,189]. MFGM and its associated proteins are present in higher concentrations in the colostrum compared to mature milk [190]. MFGM composition varies across different stages of lactation, and it is influenced by maternal factors such as body composition, diet, gestation period, infant sex, and environmental factors such as infections [189].

The lipid to protein weight ratio in MFGM is approximately 1:1, highlighting the protein presence at the MFGM [172]. Among many MFGM-associated proteins that have been identified in human milk, lactadherin, butyrophilin and mucins stand out as they are able to resist gastric digestion, and exert protective effects on the new-born [189]. MFGM-associated bioactive components may influence the microbial composition of infants, which in turn, can induce protective effects on the CNS and the immune system via the gut-brain axis [189]. For instance, MFGM proteins lactadherin and mucin-1 have antimicrobial properties and modulate the binding of bacteria in the infant gut [190]. These proteins selectively promote the growth of commensals and obstruct the propagation of pathogens in the neonatal intestine [191].

The MFGM in human milk has been linked to cognitive and health benefits in humans [171] and rodents [192,193]. Sphingomyelin, phosphatidylcholine, and phosphatidylethanolamine are all highly present components of the MFGM, with choline as a common precursor of the neurotransmitter acetylcholine which is involved in the motor neuron activity of the autonomic nervous system [194], and is implicated in CNS functions such as arousal, attention and memory [195,196]. Nonetheless, acetylcholine together with folate and betaine are essential for early life CNS development as have been discussed in Section 2.1 and Section 2.2.1. Sphingomyelins in the MFGM are especially important for myelination and have been shown to improve the neurobehavioral development of low-birth weight infants [197]. Recent studies have shown that MFGM supplementation ameliorated the visceral sensitivity and cognitive impacts of early life stress, in maternally separated rats compared to controls [193] and was able to partly improve spatial learning and memory [192]. The functional properties of the MFGM on infant cognitive and general development and health have been recently extensively reviewed elsewhere [165].

##### Human Milk Oligosaccharides and Sialic Acid

Human milk oligosaccharides (HMOs) such as 2-fucosyllactose and lacto-N-tetraose, are indigestible glycans that pass through the GI tract. HMOs composition is affected by maternal characteristics such as maternal BMI and geography [198], genetics [199] and diet [200]. In a recent study, a short-term dietary intervention in lactating mothers induced significant alterations in the HMO composition, showing that dietary choices during lactation impacts HMOs rapidly [200]. Upon reaching the intestines, HMOs are fermented by microbiota serving as prebiotics and, promoting microbial growth in a strain-specific manner [201]. For instance, *Bifidobacterium infantis* has a very specific preference to metabolize lacto-N-tetraose, but is also able to grow sufficiently in other HMOs [202], while various strains of *Bacteroides* have been found to digest only specific types of HMOs in vitro [203]. Therefore, the presence of different HMOs in human milk may induce different effects on the microbiota of each infant, depending on the strains that are already present in the gut, as well as in microbiota supplied by the breast milk [204].

More than 200 separate types of HMOs are present in maternal milk which have a protective action on the infant gut. They induce maturation of epithelial cells and improve gut barrier function, protecting the infant gut from infections and, promoting the viability and diversity of commensals [185]. A few clinical studies validated that HMOs presence promotes the growth of *Bifidobacterium* in the intestine, a genus that is abundant in the gut of breastfed infants [205,206,207]. Moreover, HMOs have protective properties against specific opportunistic pathogenic bacteria such as *Salmonella*, *Listeria*, and *Campylobacter*, by preventing those pathogens from attaching to the intestinal wall, hence forcing them to be excreted from the GI tract [184].

HMOs’ action can also improve infant health and neurodevelopment via microbiota-gut-brain axis. It has been recently suggested that there is a link between 2-fucosyllactose consumption, frequency of breastfeeding and cognitive development in 1-month old breastfed infants [187]. Moreover, HMO consumption has been linked to improved neurodevelopment and cognition in both clinical [208] and preclinical studies [209,210,211]. In rodents, 2-fucosyllactose supplementation improves hippocampal learning and memory formation [209], as well as enhances cognition all the way through adulthood [210].

HMO-associated sialic acid is another bioactive component of breast milk, that is more abundant in the early stages of lactation compared to term milk [212,213]. Sialic acid is known to promote cognition and neurodevelopment in animal models, and it is associated with gangliosides homeostasis in the CNS [212,214,215], as well as modulation of the microbiota in the gut [215]. Sialylated HMO supplementation improves learning and memory in piglets [216] and is associated with increased sialic acid concentration in brain regions linked to those cognitive changes [217]. Interestingly, sialylated HMO supplementation led to better cognitive outcomes compared to free sialic acid supplementation in rats [218]. Sialylated milk oligosaccharides are prebiotics that influence the microbiota composition in the infant [219] and the mouse intestine and reduce stress-induced anxiety like behaviour [220]. The beneficial effects of sialylated and non-sialylated HMOs present in breast milk are evident in the gut [185] and the brain [209,210,211] via the modulation of the microbiota-gut-brain axis signalling.

##### Breast Milk Microbiota

Even though breast milk was initially considered sterile, microbial metabolites were detected in the colostrum, which is the first milk produced after birth [201]. Intestinal microbial composition analysis of exclusively breast-fed neonates has shown greater abundance of *Bifidobacterium* and *Bacteroides* genera, compared to formula-fed infants [201,221,222,223,224]. This finding has led to two possible conclusions: that there is probable bacterial trafficking via breast milk to the neonate and/or that breast milk is a better substrate for particular commensals than commercial infant formula [201].

Various mechanisms have been proposed for microbiota transmission via breastfeeding such as (1) contact with maternal skin, (2) retrograde flow from the infant oral cavity to the ductal tissue and (3) enteromammary trafficking [201]. In order to investigate the first hypothesis, lactating women were advised to clean the areolar skin area before milk expression [120,225]. However, even after cleaning, enteric- and skin-related microbes were evident in the breast milk [120,225] justifying that there is another pathway of microbial flow in breast milk. The second hypothesis, called retrograde flow hypothesis, entails the flow of microbes from the infant oral cavity to the mammary gland. Even though retrograde flow of milk from the oral cavity to the ductal tissue is evident [226], certain microbes present in breast milk (such as *Actinomyces*, *Bifidobacterium* and *Lactobacillus*) are not present in the neonatal oral cavity before the onset of breastfeeding [225]. Therefore this hypothesis could justify the presence of some microbes in the ductal tissue from the neonatal oral cavity, but could not justify the complex composition of microbes found in breast milk [201]. Additionally, there are bacterial DNA traces present in the colostrum before the beginning of breastfeeding [120]. The third hypothesis, termed enteromammary trafficking includes the engulfment of intestinal maternal bacteria by mucosal intestinal dendritic cells, translocation of these cells via the circulatory or the lymphoid system to the mammary gland and, finally, transportation of microbiota via breast milk to the neonate [201]. Genomic data from three clinical studies suggest that there are common traces of *Bifidobacterium longum*, *Bifidobacterium pseudocatenulatum* and *Streptococcus thermophilus* in maternal stool, maternal blood, breast milk and infant stool samples highlighting the maternal-breast milk-infant link in the onset of infant microbiota [227,228,229]. Of the three hypotheses the latter is most plausible, as it also explains the presence of microbes already in the colostrum, even before the onset of breastfeeding [201].

Breast milk has a dynamic microbial assembly composed of mammary skin-, maternal gut- and neonatal oral-associated viable microorganisms with more than 200 different genera present [120], including *Lactobacilli*, *Staphylococci*, *Streptococci* and *Bifidobacteria* [201,225,230]. The microbial assembly of breast milk is highly influenced by maternal and infant factors such as maternal pregestational BMI, weight and diet, stage of lactation, mode of delivery as well as antibiotic exposure, infant gender and method of milk expression [121,231,232].

For instance, a maternal Mediterranean diet led to increased abundance of *Lactobacilli* compared to Western diet, in the mammary glands of a non-human primate model [232]. In another recent human study maternal diet modified the HMO composition in breast milk and the metagenomic (but not taxonomic) landscape of milk bacteria as a response to dietary-related alterations of the HMOs in human milk [200]. These alterations in milk microbiota, link maternal nutrition to metabolic capacity of milk microbiota that are seeded to the infant gut. Maternal diet during lactation changes the metabolic capacity of her gut and breast milk microbiota that are subsequently seeded to the infant gut during lactation, leaving microbial and metabolic signatures during critical temporal windows of development [200].

As long as breastfeeding is maintained, breast milk microbiota is an endless supply of colonizing bacteria for the infant gut and exert their effects via the gut-brain axis on modulation of intestinal, neurological and behavioural functions in early life that could possibly imprint the CNS and immune system throughout the lifespan [5,121,233]. Diet remains the major regulator of both maternal and infant intestinal microbiota [63] as well as breast milk microbiota [121,200]. Some breastfeeding-related protective mechanisms on infants are also partially attributed to microbiota present in breast milk itself [121].

#### 2.4.3. Infant Formula

Infant formula is a manufactured food designed for feeding children under 12 months of age. There are multiple reasons why infant formula feeding is a necessary alternative such as inability to breastfeed due to health issues or socioeconomic reasons (mother not present due to work, single parents etc). However, infant formula feeding is not recommended unless there is no other alternative, with breastfeeding being the first choice and breast milk from donors following in second place [234]. Infant formula production is highly regulated and is produced to resemble breast milk as much as possible with close attention paid to nutrient composition, bioactive components, taste and texture [60].

##### Macronutrients in Infant Formula

The macronutrient composition of infant formula has changed rapidly over the last decade. The majority of the constituent protein is derived from bovine milk, which is generally lower in quality compared to human milk proteins, primarily due to the limited amount of essential amino acids present in bovine milk [186]. The protein content of bovine-based infant formula ranges between 2–3 g/100 mL [235], which is approximately two to three times higher compared to protein present in breast milk (ranges from 1.4–1.6 g/100 mL in colostrum, 0.8–1.0 g/mL in mature milk) [161]. Although the quantity of protein is very different between human milk and bovine-based infant formula, the most abundant proteins present are quite similar; casein and whey protein with ratio fluctuating from 2:1 to 4:1 in colostrum and 1:1 in mature breast milk [166] compared with ratio ranging between 5:1 to 4:1 in bovine-based infant formula [60,235]. Apart from standardizing the protein content to 1.8 g/100 kcal in infant formula [235], companies that produce formula now add proteins with bioactive effects, such as lactoferrin [236]. However, other types of proteins have been used in infant formula to cover the nutritional needs of the baby such as goat milk protein or plant-based proteins from soy bean or green peas [186]. While the gap between infant formula and breast milk is closing, not only in terms of protein content but also regarding carbohydrates, bioactive components (such as lactoferrin, HMOs and hormones), there is still a long way to go [186]. More research is needed to evaluate the mechanisms that the various components of infant formula impact on the health of children from infancy to adulthood, compared to breastfed infants. More detailed information about macronutrient composition in infant formula can be found in Table 4.

##### Prebiotics, Probiotics and Synbiotics in Infant Formula

Health benefits of HMOs present in human milk on the intestine, the diversity of commensals, the immune and the central nervous systems incentivised infant formula-producing companies to supplement formulas with prebiotics in order to narrow the gap between human milk and infant formula [241]. Prebiotics supplementation is predominantly done with addition of short-chain galacto-oligosaccharides (scGOS) and long-chain fructo-oligosaccharides (lcFOS) which are both efficiently metabolized by *Bifidobacterium*, contrary to the HMOs present in human milk that are only partly metabolized by this genus [242]. Two other HMOs that have been more recently added to infant formula are 2-fucosyllactose and Lacto-N-neotetraoze [243]. Consumption of fortified infant formula with these two prebiotics led to a similar composition of faecal microbiota between formula-fed and breastfed infants at 3 months of age [243], but this effect disappeared by 12 months of age [187].

Prebiotic supplementation of infant formula led to significantly altered microbiota composition in infants compared to non-supplemented control infant formula [244] and has been recently reviewed elsewhere [245]. However, it is worth mentioning that counts of *Bifidobacterium* [246,247] and *Lactobacillus* [247] were found to increase with prebiotic supplementation. On the other hand, a decrease of specific pathogens (such as *Escherichia coli*, *Enterococcus* and *Clostridium*) was evident in faecal samples of 3-month-old infants receiving prebiotic supplemented infant formula compared to infants that received non-supplemented one [243,245]. Supplementation of infant formula with prebiotics led to weight gain compared to non-supplemented one, but had no effect on height or head circumference in children, which are indications of growth [244,248].

In order to maximize the advantages of prebiotics, combined prebiotic mixtures with different genera of bacteria (largely *Bifidobacterium* and *Lactobacillus* strains), have been developed in order to create infant formula as close as possible to human milk [186,249]. Even though supplementation of infant formula with prebiotics is becoming more and more common, there are difficulties in mimicking the structural and physiological complexity of HMOs [186].

Supplementation of infant formula with synbiotics (prebiotic and probiotics) is an intricate process which has difficulty in mirroring the unique combination and complexity of ingredients present in breast milk [244,249]. The prevailing ideology of synbiotics supplementation is that prebiotics can be specifically tailored for the probiotics within the same mixture, therefore the synergistic effect of the two would possibly exert health benefits that are similar to the ones of breast milk microbiota and HMOs in breast milk [244]. Most synbiotics contain a mixture of GOS and FOS together with strains of *Bifidobacterium* and *Lactobacillus* [186] (see Table 5). So far, limited clinical data collected has shown that there was no effect of synbiotic supplementation in infant formula on child development [244,250,251,252]. Hence, more research is needed to examine the possible effect of synbiotic fortification in infant formula on short- and long-term infant growth and health. Of note a promising study in rural Indian new-borns revealed significant reduction on sepsis and death levels of new-borns who were treated with a synbiotic mixture of *Lactobacillus* plantarum and FOS compared to untreated controls [253].

##### Infant Formula, Risks and Health Concerns

Infant formula-fed infants have a faster growth curve that correlates with higher weight gain, advanced adiposity, and a higher risk of childhood obesity compared to age-matched breastfed infants [201]. Interestingly, infant formula consumption leaves a signature on glucose metabolism and insulin sensitivity, that persists to adulthood [254]. High insulin levels in formula-fed infants and its respective advanced adiposity in early life are correlated with high protein levels (2.9–4.4 g/100 kcal) found in high-protein infant formulas [255]. The timing at which formula is introduced to the infant is also a determinant of health in the neonatal period and adulthood; introduction of infant formula before the third month of life is correlated with increased risk of rapid growth at six months and high body mass index in adulthood [256]. Allergies are also prevalent among infants consuming infant formula, mainly due to sensitivity against casein or β-lactalbumin present in bovine milk [60].

Neonatal nutrition is a determining component of health in the early days and its effect remains throughout life. Improvement of structural and functional properties of formulas to minimize the differences between breastfed and formula-fed infants is strongly supported by research on infant formula. More independent research is important to reach conclusive results regarding the short- and long-term effects of the components of infant formula on human health, as well as the causation of the differences between formula-fed and breast-fed infants [186]. It is important to understand and distinguish correlation and causation, as well as the complexity of factors influencing these early life experiences.

## 3. Microbiota Changes and Neurodevelopment: From Infancy to Childhood

### 3.1. Microbiota Maturation and Brain Development during Weaning

In humans, the gradual introduction of solid food and the progressive termination of breastfeeding starts between 4–6 months after birth and is called weaning [257]. During this period, in order to cover the nutritional needs of the baby, parents might choose to complement the diet with infant formula or introduce solid food progressively. Solid food introduction is a major modifier of microbiota composition in the gut and it dictates the beginning of intestinal microbiota maturation [258]. This microbiota maturation is gradual, reaching a more diverse, yet stable composition [245].

Introduction of solid foods during the transition from a milk-based to a more complex adult-like diet, changes the richness and diversity of the microbiota landscape [245,258]. During this change in food substrates, counts of milk-related bacteria are reduced and genera able to digest more complex nutrients are expanding. For instance, certain genera such as *Lachnospiraceae*, *Ruminococcaceae, Blautia, Bacteroides*, and *Akkermansia* are blooming [258], while *Bifidobacterium*, *Veillonellaceae, Lactobacillaceae, Enterobacteriaceae*, and *Enterococcaceae* counts are gradually decreased [258]. As long as the early feeding mode (breast- or formula feeding) continues to be present, the microbiota landscape displays an in-between state of the infant and the adult compositions [34].

Throughout those substrate changes during weaning, the microbiota’s metabolic capacity also shifts in the infant gut with increasing genes linked to complex polysaccharide metabolism such as starch [259]. Nutrition is a major modulator of microbiota colonization and propagation in the intestine [63], and therefore, their respective microbial signals are crucial for healthy development of neuronal circuits in the brain, as well as for behavioural imprinting [9]. The nutritional changes during the solid food introduction period coincides with the sensitive developmental period around weaning (see Figure 2). However, more clinical studies are needed to investigate the microbial changes during weaning and their long-term effects in humans.

Increased emphasis is being placed on the role of the microbiota in gating immunological changes to weaning [164]. The implications this has for brain function is currently unknown. However, given that microbiota maturation in the infant gut coincides with postnatal brain development [9,59] such as synapse propagation and pruning [78] it is highly likely to be relevant. Neurons are highly plastic in response to environmental stimuli during windows of opportunity, such as the postnatal period, resulting in irreversible changes that persist throughout life [79]. It is hypothesized that the intestinal microbiota has a role in structural and functional aspects of brain development and maturation [42,106]. Indeed, the total absence of microbiota in GF animals led to structural alterations in the brain, with GF animals having lower density and length of dendritic spines as well as enlarged amygdala and hippocampi compared to conventional mice [260].

### 3.2. Solid Food Introduction and Establishment of Nutritional Habits

Dietary habits of the mother during pregnancy and food choices early in life are known to be essential for the establishment of odours and taste preferences in later life [261]. The olfactory system, which includes the olfactory bulbs in the brain that are responsible for taste and smell perception, is functional already at the 24th week of gestation [262]. The amniotic fluid is full of nutrients from maternal diet and stores and, it is the first ‘food’ the fetus is able to detect different tastes and flavours from [263]. Behavioural mechanisms, such as programming of food preferences and eating behaviour start very early in life and have been recently discussed elsewhere [264].

During the initial years after birth the seeded microbiota continues to develop and mature with multiple factors such as nutrition, environmental and maternal factors, dictating its propagation in critical temporal windows of development. For instance, perinatal factors such as delivery mode or gestational age at birth have been recently unravelled as determinants of the gut microbiota composition up to four years of age in humans [118]. These findings suggest that events that disturb normal microbial seeding perinatally might leave signatures in the first steps of life, and it is therefore hypothesized that early life microbial perturbations possibly prime developmental processes which tip the balance between health and disease.

## 4. Early Life Microbiota: Another Component in the Vicious Cycle of Malnutrition

Malnutrition is an umbrella term and in its whole spectrum includes undernutrition (wasting, stunting, underweight), overweight and obesity and remains a global health challenge up until today [265]. Apart from extreme undernutrition, moderate caloric and nutrient restriction during pregnancy (self-imposed maternal dieting or teenage pregnancy) is common in developing and developed countries [266]. The consistently decreased exposure to proper quantity and quality of food during pregnancy can lead to irreversible changes in crucial body systems such as the CNS [267], cardiovascular [268], haemopoietic [269] and immune system [270] of the developing fetus.

The nutrient availability for the developing fetus depends on the concentration of those nutrients in maternal circulation and the blood flow in the placenta that will distribute them to the embryo [271]. After birth, the most prominent factors for the onset of severe acute early life malnutrition are the abnormal or early cessation of breastfeeding, premature weaning and low-quality of breast milk due to disturbed maternal nutritional and general health status [272].

### 4.1. Undernutrition and CNS Development

During pregnancy, even mild or moderate caloric restriction could have a dramatic impact on the fate of CNS development. In a non-human primate model, moderate caloric restriction in the mother led to decreased cerebral development, neurotrophic factor suppression, impaired axonal growth and glial maturation in the offspring [273]. In the same study, decreased maternal nutrient availability resulted in downregulation of transcriptome pathways related to brain development and cell proliferation of the embryo, while pathways related to cerebral catabolism and cell death were significantly upregulated [273].

In rodents, reduced maternal protein intake during pregnancy (5 or 10% protein as compared to 20% protein) caused not only impairments in brain development and growth rate of the pups, but also in protein and fatty acid metabolism, in the brain and on a whole-body level [274]. In another similar rodent study, dam exposure to a low-protein diet during pregnancy through 4 weeks postnatally, resulted in decreased *BDNF* gene expression levels in the hippocampus of the offspring accompanied by impaired spatial learning and memory [275]. Moderate nutritional/caloric restriction during pregnancy impacts brain metabolism [274], and gene and protein expression of key-regulators for CNS health, and affects cerebral development of the embryo which leads to behavioural adversities later in life, in preclinical models [275].

In humans, insufficient early life energy, protein iron and iodine intake leads to impaired behavioural and cognitive function that persist to adulthood. Deficiency of B vitamins, zinc, betaine and choline are able to induce irreversible changes in the brain and lead to neurological deficits on the developing child [87,276]. During the infamous period of the Dutch famine in the mid-1940s, when exposure to nutrients was widely insufficient, the malnutrition cases were extremely high [276]. Birth cohorts and later follow-up studies showed that pre- and perinatal malnutrition increased the prevalence of congenital disease of the CNS in early life, schizophrenia and schizophrenia spectrum personality disorders in adults that were exposed to the famine as infants. Malnutrition or undernutrition during crucial developmental time-windows, for instance during synapse formation, could lead to impaired brain growth with no or possible minimal recovery after nutritional rehabilitation at a later time-point [86,277]. Recovery is possible only after nutritional rehabilitation before the end of the critical period of synapse formation, proliferation and brain growth [278].

### 4.2. Undernutrition and Microbiota

Optimal nutrition in early life is crucial for the expansion of the microbiota, immune system development, and the balance of host-microbe interactions. Exposure of 3-week-old mice to reduced dietary protein resulted in decreased binding of intestinal immunoglobulin A (IgA) with commensal bacteria, interfering with normal mucosal processes in the mouse intestine [63]. This disrupted relationship between IgA and gut commensals led to adaptation of bacteria to nutrient limitation and modulated the host-microbial homeostasis by reducing the binding of commensals on the intestinal mucosa [63]. In another similar preclinical study, dietary intervention with tryptophan-deficient diet in mice led to vitamin B3 deficiency which modulates the production of antimicrobial peptides in the intestine [279]. This dietary intervention led to altered microbiota composition accompanied by inflammatory colitis attributed to the microbial composition changes [279]. Therefore, postnatally and during time-windows of opportunity such as weaning, undernutrition regulates important homeostatic host-microbe interactions.

In severely malnourished children it is difficult to identify whether the changes in gut microbiota composition present as a consequence of the diet or the condition or the combination of both [272]. A common complication in severely malnourished children is enteric septicaemia with increase prevalence of *Salmonella*, *Shigella* and *Staphylococcus aureus* populations in faecal samples [280]. These bacteria were able to grow in blood cultures, suggesting that in the presence of a leaky gut, a common outcome in undernourished populations, the pathogens possibly survive and migrate to other previously sterile tissues via the systemic circulation [280].

Microbiota composition in malnourished children is disturbed with almost depleted *Bifidobacterium* and an inverted aerobes:anaerobes ratio in faecal samples resembling a non-mature microbiota [181,281]. Nutritional intervention alone is unable to inverse the unhealthy aerobes:anaerobes ratio, suggesting that diet alone is unable to modulate the unhealthy microbiota after it has been established in critical temporal windows during early life [281]. Interestingly, antibiotic treatment with cefdinir and amoxicillin has shown promising results in systemic treatment of malnourished children, by decreasing the incidence of diarrhoea and mortality while promoting weight gain [280]. Antibiotic treatment accompanied by therapeutic diet and probiotic supplementation with *Lactobacillus delbrueckii* effectively reduced mortality of malnourished children [280]. The combination of antibiotics together with high-quality protein and high-fibre diet (prebiotic) as well as supplementation with probiotic bacteria might be a more targeted approach to malnutrition [272].

Recent approaches have focused on targeting the microbiome for undernourished children via societal strategies such as educational support highlighting the importance of the microbiota on high-risk communities [282] and promotion of microbiota-directed complementary foods that aim at the maturation of the stunted-like, immature microbiota, present in the intestine of those individuals [282,283]. Even though studies on undernutrition usually focus on general child development, links between microbiota and brain developmental changes induced by malnutrition have also been highlighted [61].

Poverty and food insecurity are major risk factors for malnutrition [284]. However, malnutrition is a complex condition and very difficult to reproduce in controlled, hygienic environments [285]. It is hypothesized that apart from the decreased nutrient availability, severe acute malnutrition has a microbial component [285]. The decreased availability of nutrients might lead to altered microbiota composition and increased inflammation in the gut that subsequently modulates the intestinal environment, the absorption of nutrients and magnifies the microbial dysbiosis in the gut [282,284]. Therefore, the vicious cycle of malnutrition has another intestinal microbial component that could partly act as a target for prevention of symptoms of the disease.

## 5. Conclusions

Microbes are co-existing and co-evolving with humans across the lifespan and it is believed that the beginning of this mutualistic relationship starts at birth. However, increasing data has shown that microbial signatures modulate embryonic development, imprint the CNS and the immune system and fine-tune the balance between health and disease. Both neurodevelopment and microbial composition in early life are plastic and influenced by parental (genetics, diet, internal stores, pathophysiology of the parents, delivery mode, feeding mode, establishment of nutritional habits) and environmental (infections, antibiotics, pollution) factors.

Early life nutrition plays a central role in the onset of multiple developmental processes in the brain and the alimentary canal via nutritional programming and modulation of the microbiota-gut-brain axis during the first steps of life, regulating the equilibrium between health and disease later in life. Understanding the link between the triad of perinatal nutrition, neurodevelopment and gut microbiota is of great importance in order to unravel the mechanisms of diseases that are believed to be rooted in early life. Although important data has recently described some of the early life nutritional programming and the microbial contribution on the imprinting of the CNS, principal mechanisms remain elusive. Perturbations that challenge the balance of this relationship during critical temporal windows of development might disturb gut homeostasis and host-microbial interactions, as well as structural and functional alterations in the CNS. More research is essential on the impact of nutrition on the shaping of the gut microbiota and neurodevelopment of the offspring to untangle the intertwined pieces of this complex puzzle that is microbiota-gut-brain axis and, to possibly prevent, diagnose and mitigate conditions that are rooted in this sensitive period.

## Figures and Tables

**Table 1:** Main components of human milk on the three stages of lactation and the effect on infant health. The cessation of breastfeeding is specific to each mother-infant dyad; it is decided according to the needs of the infant and upon the choice of the mother.

Stages of Lactation	Duration	Components	Effect on	References
Stage 1: Colostrum	1–5 days postnatally	Very rich in immunoglobulins, lactoferrin, leukocytes, growth factors, vitamins A and E, proteins and fat low quantities of lactose	Immune system development	[147,151]
Stage 2: Transitional milk	1–2 weeks postnatally	Richer in lactose and fat compared to colostrum, richer in proteins and fat compared to mature milk	Nutritional needs of the baby	[147,151]
Stage 3: Mature milk	1st month–end of lactation	Richer in lactose and water compared to colostrum and transitional milk	Nutritional needs of the baby	[151]
		Richer in vitamins B1 and B6 compared to colostrum and transitional milk		[147,151]

**Table 2:** Macro and micronutrients present in human milk, their effect on infant health and their expression in different stages of lactation.

Category of Molecule	Breastmilk Component	Subcategory of Molecule	Effect on	References	Population	Highly Present in which Stage of Lactation
Macronutrients	Casein	Proteins	Immune system development, general growth and development	[166]	Humans: multiple	More abundant in colostrum compared to mature milk
	α-Lactalbumin			[167]	Humans: multiple	
	Lactoferrin			[168]	Humans: multiple	
	Immunoglobulins			[169]	In vitro, mice & humans	
	Lysozyme			[147]	Humans: multiple	
	Serum albumin			[166]	Humans: multiple	
	Palmitic and oleic acid	Heterogenous mixture of proteins, lipids, fatty acids and cholesterol	General growth and development	[170]	Humans: multiple	Richer in colostrum compared to mature milk and richer in evening compared to morning feedings
	Milk fat globule membranes: MFGM		Cognition, neurodevelopment, boost immune system against infections	[165,171,172]	In vitro, mice and humans	
	Lactose	Carbohydrates	Growth and development	[147]	Humans: multiple	More abundant in mature milk and when milk is expressed more frequently
Micronutrient	Vitamins A and E	Vitamins	Growth and development	[147,151]	Humans: multiple, mice and rats	Higher in colostrum compared to mature milk
	B complex vitamins	Micronutrients	Epigenetic potential, neurotransmitter synthesis, neurodevelopment, protection from neural tube defect	[15]	In vitro, mice and humans	B1 and B6 higher in mature milk compared to colostrum
	Iron, copper and zinc	Metals	Neurodevelopment, haematopoiesis	[147,151]	In vitro, mice and humans	Higher in colostrum compared to mature milk

**Table 3:** Bioactive components of human milk and their effect on infant health.

Breastmilk Component	Subcategory of Molecule	Effect on	References	Population	Stage of Lactation
**Epidermal growth factor (EGF)**	Growth factors	Intestinal maturation, immune system imprinting during weaning	[164]	Mice	Present as long as the lactation lasts
		Enterocyte stimulation for nutrient absorption	[173]	Pigs, human	
		Tight junction and cell death regulation in response to gut inflammation	[174]	Rats	
**Brain-derived neurotrophic factor (BDNF)**		Normal growth, development and function of neurons in the CNS* and PNS*	[175]	Rats	Present in breast milk up to 90 days postnatally
		Increase intestinal motility by stimulation of the ENS*	[176]	In vitro and rats (*ex vivo*)	
**Glial-derived neurotrophic factor (GDNF)**		Normal growth, development and function of glial cells in the CNS* and PNS*, supports neuronal health and development	[175]	Mice and rats	
**Erythropoietin (EPO)**	Hormones	Protective effect on intestinal tight junction, prevent anaemia and reduces the risk of necrotizing enterocolitis	[177]	In vitro	
**Adiponectin**		Regulates metabolism and suppresses inflammation	[178,179]	Humans: Hispanic, mice and humans, humans: Hispanic	
		Regulates body weight later in life	[178,179]	Mice and humans, humans: Hispanic	
**Ghrelin and leptin**		Control appetite, body composition and metabolism	[180,181]	Humans: multiple, Humans: Caucasians, humans: multiple	
**Prolactin**		Stimulating milk production	[151]	Humans: multiple, mice and rats	
**Oxytocin**		Production stimulated in PVN* by skin to skin contact with the mother	[151,182]	Humans: multiple	Low levels in breast milk
		Sociability	[183]	Mice and Humans: multiple	
**Breast milk microbiota**	Microbiota	Modulate the gut-brain axis, boost gut barrier function, improve the development of intestinal diseases, are able to rescue behavioural deficits, as well as anxiety-like and depressive-like behaviour, in preclinical models, regulate cytokine and tryptophan levels in mice, shape neurodevelopment, promote synaptic formation and microglial action	[141,142,143]	Rats, mice, humans: multiple, mice	Through the course of lactation
**Human-milk oligosaccharides (HMOs) (lacto-N-tetraoze, 2-Fucusyllactose)**	Carbohydrates + prebiotics (concentration varies depending on stage of lactation)	Modulate the microbiota-gut-brain axis	[151,184,185,186,187]	In vitro, mice, rats and humans: multiple	Through the course of lactation
		Protect from infection in the gut by reducing colonization of pathogens and promoting the viability and diversity of commensals	[184]	Mice, rats and humans: multiple	
		Improve cognitive development	[187]	Humans: Hispanic	
		Inducing maturity of epithelial cells and improve gut barrier function	[151,184,185,186,187]	In vitro, mice, rats and humans: multiple	
**Macrophages**	Cells	Protection against infection, T-cell activation	[169]	Humans: Indo-Aryan and In vitro, mice	More abundant in colostrum than mature milk
**Stem cells**		Regeneration and repair	[188]	Humans: Caucasian, Indo-Aryan	

CNS*: central nervous system, PNS*: peripheral nervous system, ENS*: enteric nervous system, PVN*: paraventricular nucleus.

**Table 4:** Macronutrients in infant formula and their effect in infant health.

Category of Molecule	Formula Component	Effect on	Associated with	References	Population
Protein	Whey protein and casein (cow-based formula)	General growth and development	Weight gain in infancy and higher adiposity later in life	[237]	Humans: Caucasian
			Allergies in milk protein	[60]	Humans: multiple, mice and rats
			Higher secretion of insulin due to stimulation of β-pancreatic cells by amino-acids	[237]	Humans: Caucasian
Fat	Mixed vegetable oils (DHA & ARA)	Adipocyte stimulation and fat storage	High consumption of ω-6 fatty acids might induce adiposity in early life	[186,238]	Humans: multiple, mice and rats
	MFGM (cow-based formula)	Fat storage and general metabolism	Decreased fat accumulation, concentrations of leptin, glucose and lipids in plasma	[239]	Mice
		Cognitive development	Improves cognition of formula-fed infants	[171]	Humans: Caucasians
Carbohydrates	Lactose	General growth and development	Low gastrointestinal tolerance	[240]	Humans: multiple

**Table 5:** Components of infant formula in order to simulate the synbiotic potential of human milk.

Category of Molecule	Formula Component	Effect on	Associated with	References	Population
HMOs	Short-chain galacto-oligosaccharides (scGOS) and long-chain fructo-oligosaccharides (lcFOS)	Highly metabolized by Bifidobacteria, contrary to the HMOs present in human milk that are only partly metabolized by Bifidobacteria	Weight gain but had no effect on height or head circumference, significantly altered microbiota composition in infants	[242]	Humans: multiple
	2-fucosyllactose (2’FL) and Lacto-N-neotetraoze (LNnT)	Similar composition of faecal microbiota between formula-fed and breastfed infants at 3 months of age		[243]	Humans: multiple, mice and In vitro
Synbiotics	GOS and FOS together with strains of *Bifidobacteria* and *Lactobacilli*	No effect on child development		[250,251,252]	Humans: Asian, Caucasian

## Data Availability

No data are produced in this study, it is only reviewing of existing data.
